# Let us do better: learning lessons for recovery of healthcare professionals during and after COVID-19

**DOI:** 10.1192/bjo.2021.981

**Published:** 2021-08-19

**Authors:** Esther Murray, Kenneth R. Kaufman, Richard Williams

**Affiliations:** Barts and the London School of Medicine and Dentistry, Queen Mary University of London, UK; Rutgers Robert Wood Johnson Medical School, New Jersey, USA; and Department of Psychological Medicine, Institute of Psychiatry, Psychology & Neuroscience, King's College London, UK; Welsh Institute for Health and Social Care, University of South Wales, UK; Royal College of Psychiatrists, UK; and Faculty of Pre-Hospital Care, Royal College of Surgeons of Edinburgh, UK

**Keywords:** COVID-19, well-being, psychosocial care, mental healthcare, primary stressors, secondary stressors, moral injury

## Abstract

The COVID-19 pandemic emphasises the need to rethink and restructure the culture of healthcare organisations if we are to ensure the long-term well-being and mental health of healthcare provider organisations and their staff. In this paper, we recognise the high levels of stress and distress among staff of healthcare services before the COVID-19 pandemic began. We identify lessons for care of healthcare staff and illustrate the paths by which support mobilises and later deteriorates. Although this paper focuses on NHS staff in the UK, we contend that similar effects are likely in most healthcare systems.

‘Do the best you can until you know better. Then, when you know better, do better.’

Maya Angelou

The core theme in this paper is that, despite all we have learned from prior disasters and infectious disease outbreaks, the COVID-19 pandemic emphasises the need to rethink and restructure the culture of healthcare organisations if we are to ensure the long-term well-being and mental health of healthcare organisations and their staff. It is essential to recognise that there were high levels of stress and distress among staff of healthcare services before the COVID-19 pandemic began. Many people, and particularly those who had prior interests in the well-being and mental health of healthcare staff, were mobilising support for staff.

The authors of this paper draw together what we knew and what we have learned from the pandemic, and identify changes in ways of caring for staff during the pandemic that should not be lost when it is eventually resolved. We think it likely that the impact of COVID-19 on the world and its healthcare systems will continue well into the future. This article focuses on experiences in the UK to illustrate the themes that we identify, although we believe that they have international merit albeit with important cultural nuances. We started writing in January 2021 as the second wave was raging in the UK, the numbers of cases of COVID-19 and deaths were at their highest as a result of the SARS-CoV-2 variant B.1.1.7, and other variants were emerging worldwide. In some parts of the UK, there was no apparent respite between waves. Now, in May 2021, we hope that we might also look back to see early spring 2021 as a turning point in the pandemic, as vaccine roll-out is well-advanced in the UK. Numbers of COVID-19 cases have dropped hugely in the UK, which we see as reflecting the success of the vaccination programme and lockdown procedures that separate people from the virus. Consequently, the UK is emerging gradually from the most restrictive measures. More doses of vaccine have been ordered as we await a potential third wave later in 2021, and we hope that another lockdown will not be required. But the pandemic continues to spread rapidly elsewhere in the world. Delays in vaccine availability worldwide are resulting in delay in resolving the pandemic, leading to more variants arising. The B.1.617 series is circulating across the world (chiefly the B.1.617.2 variant in the UK).

We emphasise that nothing in this paper should be construed as taking away any acknowledgement whatsoever of the enormously positive work done by the National Health Service (NHS) as a whole, and by all its staff in the most challenging of circumstances. But we do forthrightly explore the nature and complexity of the challenges, with a view to learning lessons for the future. What comes across is the vital nature of integrating planning for sustaining the well-being, psychosocial care and mental healthcare of all staff within all plans for emergencies.

## Background

A recent systematic review of healthcare workers at risk of distress during an infectious disease outbreak has identified evidence that their distress can persist for several years.^1^ That review covered 138 papers predominantly about COVID-19 and severe acute respiratory syndrome (SARS); an additional ten papers addressed Middle East respiratory syndrome (MERS), influenza strains H1N1 and H7N9, and Ebola. The review identifies consistent evidence that being female, a nurse, experiencing stigma, having contact or risk of contact with infected patients, and experiencing quarantine were risk factors. Personal and organisational social support, perceived control, positive work attitudes, sufficient information and proper protection, training and resources were associated with less distress. Earlier in the pandemic, Kisely et al reported their systematic review of the psychological effects of emerging virus outbreaks on healthcare workers over the past 20 or so years.^2^ They concluded that the organisational measures that best decrease the risk of adverse outcomes include positive feedback to staff, the faith of staff in local infection control procedures, providing protective gear, effective preparation and training. There should be protocols for supporting staff, clear communication with them and access to tailored psychosocial interventions based on needs identified by members of staff.

Reading this list, it is easy to see that many of these factors are highly likely to be protective of healthcare staff well beyond serious outbreaks such as pandemic flu, HIV, SARS, Ebola, MERS and COVID-19. But many people who work in healthcare know that these features and the working conditions of healthcare staff are too rarely given adequate attention in any healthcare setting.^3^ Access to psychosocial care and interventions based on the needs of groups of staff is not always straightforward, nor does it form part of the culture of many organisations.

### Legacy problems in the UK

In the run-up to the COVID-19 outbreak, there was wide recognition that the workforce of the four national health services in the UK (hereafter, the NHS) and healthcare staff were chronically stressed, overstretched and under-resourced.^3^

In the UK, these problems have been described as the pre-COVID-19 legacy. However, over the course of some years, a movement consisting, mainly, of staff had been growing; it looked to address the matter of staff well-being and early intervention for staff who were struggling through, for example, stress management workshops, mindfulness training, provision of yoga and arts and crafts classes, and creative enquiry.^4–6^

Other aspects of working life that inspired staff to find their own solutions were, for example, the rota system; during the pandemic, self-rostering was rolled out in some hospitals to give staff better control over their working lives.^7^ Many interventions were delivered by the staff themselves, who knew the problems in their services and sought to address them, usually at no cost, on hospital premises and, only rarely, in protected time such as during audit or clinical governance days. There were few psychological and social interventions, and most of the interventions delivered were not formally evaluated, meaning that there is little in the way of an evidence base. Welfare ambassadors and psychological first aiders had begun to appear in some hospital departments. but their presence relied on the voluntary efforts of staff.^8^

It is a testament to the commitment of staff that so many ‘home-grown’ interventions were created, resulting in a strong movement of NHS and other healthcare staff in the UK delivering workshops, creating material for websites and maintaining an active social media presence in the arena of staff well-being. In many healthcare organisations, senior managers have become advocates of paying due care and attention to staff well-being, although the majority of the activity still seems to come from staff via arranging study days and the like through their respective collegiate organisations.

Nonetheless, when the pandemic hit, an already stressed (and in many cases, chronically distressed) workforce was required to face, and has stepped up to, a challenge for which it was ill-equipped and ill-prepared. Yet, there was enough awareness of the psychosocial impacts of working in healthcare, and enough evidence about the psychosocial impacts of major incidents, pandemics and epidemics, to alert healthcare organisations to the fact that care and support would be needed.^9^ Although we might make observations about the speed at which support was provided during the first wave, we can see that there is a new attitude to the risks of working in healthcare, especially in a pandemic, and an understanding that ‘psychological’ personal protective equipment (PPE) is as important as physical PPE.^10^ Given our understanding of what works in this situation, and the increasing body of knowledge from psychology and the front line of NHS care underpinning what works for healthcare staff in more usual times, we would do well to take the lessons from major incidents and COVID-19 forward into a rapidly evolved way of leading healthcare professionals and managing healthcare services. It is clear that we are at risk of misunderstanding the challenge if we think that the arrangements are likely to return to how they were before COVID-19.

Now, in May 2021 in the UK, the situation continues to develop. Although daily news bulletins report that the number of people being treated for COVID-19 is massively reduced, staff are now telling us anecdotally about the huge fatigue they are experiencing, large numbers of nurses are said to be reporting sick and a not inconsiderable number are leaving their jobs. Key is likely to be nurses leaving their jobs; this will result in increased stress secondary to understaffing, further compounding the difficulties of successful recovery. Thus, the most pressing current challenge is recovery. As vaccines are rolled out in the UK and there is more talk from central government and the media of a ‘return to normal’, healthcare staff are the subject of two conflicting narratives: that there will be a ‘recovery’ programme for them, but also that they will address the enormous backlog of patients whose treatment was suspended or delayed by COVID-19 (i.e. the functioning of the NHS will recover). We will be living alongside COVID-19 for a long time to come, and the resources to manage it and deal with waiting lists are not necessarily in place. We discuss issues related to this scenario later in this paper.

We argue that the goal is not only recovery once the ravages of the second and any succeeding waves have diminished, but also learning lessons for new ways of working together.

## Stress and healthcare staff

To focus on what needs to change, we must consider the stresses that affect staff who work in healthcare services.^11^ The major worries that have stood out in the world context and in the UK, particularly during the first wave of the pandemic, include, but are not limited to,^12^ worry about contracting the disease, worry for staff about conveying the disease to family members, worry about making a mistake, inadequate PPE, inadequate testing, staff from Black and minority ethnic communities and backgrounds have been identified as being at particular risk of contracting COVID-19,^13^ and failings to sufficiently protect staff from Black and minority ethnic communities and backgrounds.^13^

Additionally, short isolation periods for people who have symptoms and failure to recognise post-viral fatigue and feelings of anxiety and depression have been other sources of concern. This list was gleaned from published papers and gives some examples of the major concerns of staff. Perhaps, it is narrow, but we are awaiting peer-reviewed articles on the topic to further expand it.

The systematic review and meta-analysis of 59 papers reported by Kisely et al concerns the occurrence, prevention and management of the psychological effects on healthcare staff of emerging virus outbreaks.^2^ Most of the papers reviewed report research on SARS, influenza caused by the H1N1 virus, MERS and Ebola, but eight papers report on the effects of COVID-19. Overall, they conclude that the risk factors for distress include being younger, being more junior, being the parents of dependent children, having an infected family member, longer quarantine, lack of practical support, and societal stigma.^2,14^ They point to the importance of interventions based on the principles of psychological first aid,^15^ and include dealing with basic needs, empathic listening, access to information, practical care and support; they do not include psychological debriefing that focuses on traumatic experiences. Kisely at al point out that the World Health Organization and United Nations emphasise preventative measures and mental health.^16,17^

As a result of the complexity of factors in emergencies and disasters, it has become common to endeavour to distinguish primary and secondary stressors. The latter may directly affect how people respond to events, but also moderate the effects of primary stressors.

### Primary stressors

Primary stressors are the sources of worry or anxiety that stem directly from the events and causative agents and the consequential tasks that face healthcare staff.^18^ They may reflect single events, but are more often an accumulation of pressure over time. They include decisions that staff believe are morally and/or professionally unfair. Hence, moral distress and moral injury are particular primary stressors of considerable current concern that have raised substantial interest during the pandemic.^3^

The COVID-19 pandemic has caused widespread concern about two major matters. First is the risk of being infected. This has been experienced as a particular stress for healthcare practitioners, many of whom have fallen sick as a result of their contacts with patients, and too many have died. This led to recognition that practitioners from Black and minority ethnic backgrounds are at particular risk of contracting the infection, and the reasons appear to be multifactorial.^13^ We construe this as a risk factor and, therefore, as an important feature of the primary stressor. A second primary stressor is the risk of healthcare staff transmitting the virus to family members and colleagues, and we recognise that this has weighed heavily on staff of healthcare services. By contrast, the problems involved in adequately protecting staff lie within individual countries’ responses, and are thus secondary stressors. Arguably, in the light of prior pandemics and national exercises of emergency preparedness, this also reflects countries not being adequately prepared in advance of COVID-19; thus, not having sufficient supplies of PPE is clearly a secondary stressor.

#### The moral effects of COVID-19

A particular aspect of this long-lasting emergency concerns the moral effects of COVID-19 on staff who are struggling to provide care for patients in circumstances that are not ideal. Thus, concerns about the effects of moral injury and moral distress on healthcare professionals have been high in the pandemic. Moral distress has long been written about in relation to healthcare, usually in relation to nurses and largely about the difficulties of delivering the care that they would like to deliver.^19^ In a pressured system, staff are challenged to be able to spend enough time with each patient to deliver what they perceive to be optimal care, which causes them great distress. Litz et al postulate that there is a point at which moral distress becomes moral injury.^20^

We note that, in the 15 months during which we have lived with COVID-19 so far, discussions of moral injury have become more frequent, and this concept is now recognised by staff of some intensive care units, for example. Although the concept of moral injury came from clinical work with military veterans in Veterans Affairs hospitals, its application to other settings is increasing. Shay describes it as ‘a betrayal of what's right, by a person in legitimate authority in a high stakes situation’.^21^ What is important in considering the role of moral injury in the time of COVID-19 is that the effects of moral transgressions, whether our own or of others, disrupt our relatedness to other people through our feelings of shame, guilt and anger. Litz et al point out that the feelings of shame and guilt might be associated with acts we have perpetrated, and feelings of anger might be associated with those acts perpetrated by others.^20^ They suggest that moral harms reduce the sense of safety of ‘us’ because, if people like us do things like this, then do I want to be part of ‘us’ anymore? *The Guardian*, a UK newspaper, reported that staff were planning to resign their posts in some of the hardest hit areas in the UK.^22^ Consider also, the outrage in the USA when a president did not reveal the dangers associated with the pandemic, and the need for specific behavioural precautions with more than 33.2 million cases and over 593 000 COVID-19-related deaths as of 29 May 2021.^23^

Discussions with staff reveal that actual morally injurious events were not always those they expected. In the period just before COVID-19 hit in the UK, and during the initial lockdown, staff were concerned about the lack of PPE, and the potential of rationing of care based on lack of resources as time passed.^24–27^ These concerns resurfaced in the second wave. During the first wave, the issues of disproportionately high deaths in certain communities, such as Newham in East London, and in certain ethnic and professional groups became hugely problematic. Other issues that arose were the disruption to care caused by PPE, which masks faces, disrupts body language, makes movement more awkward and muffles voices.^28^ Staff have to use PPE to keep themselves and patients safe, but it affects communications in healthcare environments, and Hampton et al call for efforts to seek alternative communication paradigms.^27,28^

Visitors to patients in hospital with COVID-19 are either not allowed or they are limited to only one or two immediate family members. Previous research addressing a vancomycin-resistant enterococcus outbreak has reported on the issue of nurses feeling that they are the gatekeepers for contacts with the sickest patients, and they struggle to deal with family members who want to visit but cannot.^29^ These findings resonate with findings from a recent survey of nurses in the UK during the COVID-19 pandemic that emphasised communication difficulties when using PPE, and distress associated with end-of-life care in the context of limitations on visits by family members.^30^ This situation compromises the quality of care the nurses and other staff want to give, and because the experience of patients dying ‘alone’ was highly aversive. Media reporting of the issue has been unhelpful. In fact, patients did not die alone, but have competent and caring staff around them; this fact does not detract from the emotional impact of ‘the wrongness of things’. The business of breaking bad news, for which all healthcare professionals receive training, is also disrupted in the COVID-19 pandemic. In most cases, it is not possible to share upsetting news with relatives face to face in the most acute phases, and this work is generally done over the telephone. Consequently, new training courses have been developed including a course to support staff in this task.^31^

### Secondary stressors

By contrast with primary stressors, secondary stressors are more diverse. A group of researchers in the field argues that most secondary stressors are a function of social factors and people's life circumstances, including the policies, practices and social, organisational and financial arrangements that affected them before the emergency and/or societal and organisational responses to the incident.^4,18,32^ Thus, secondary stressors also include the adequacy and effectiveness of employers’ responses to the primary stressors and their expectations of employees’ performance.

Chief among the secondary stressors in the pandemic is inadequate PPE and the changing advice about what protection to use in which circumstances, because these matters both reflect how healthcare organisations responded to the emergency. They rapidly became a major concern during the first wave, but also resurfaced in the second wave. The speed and frequency at which recommendations regarding PPE changed during the first wave resulted in junior staff feeling stressed about whether the information they were given was correct, and senior staff feeling stressed about passing on advice regarding PPE that they believed to be inappropriate. During the second wave, concerns were voiced again about the adequacy of the PPE provided for the most acute settings and for staff of ambulance services.

As we have seen, the matter of PPE is complicated. Working while wearing it exacerbates some primary stressors, but working without it is extremely risky. We have illustrated how problems with supply and wearing PPE resonated into relationships with patients and the moral experiences of staff. Plainly, perceptions of inadequate PPE interacted with, and amplified, the worries that staff have about being infected or transmitting infection at work and at home. Thus, secondary stressors often exacerbate or moderate the effects of primary stressors.

Our anecdotal enquiries suggest that there have been many other problems in the world's responses, including those in the UK, that were intended to diminish the primary and secondary stressors that affect healthcare staff. Some secondary stressors relate to the pre-pandemic circumstances, including the computer infrastructure and bureaucracy that has been problematic for some time. In February 2021, for example, as doctors rotated to different hospitals, their computer accounts were not properly reallocated, leaving them locked out of areas of the hospitals and computer accounts while trying to run an intensive care unit full of patients with COVID-19. There have been widespread problems with salary payments, including regular payments and COVID-19 top-up payments for bank work. Secondary stressors also include the career aspirations and concerns of staff about their training, the conditions in which they work and live, and their work–life balance. All of these matters reduce the effectiveness of staff and their perceptions of themselves as helping others.

These examples illustrate the variety of mechanisms by which secondary stressors operate; they include direct interactions with primary stressors and also indirect interactions, through reducing the morale, well-being, attention and cohesion of teams. Thus, recurrently, staff of public organisations have said that secondary stressors affect them to a greater degree than primary stressors, and the matters raised by staff who seek help very frequently concern secondary stressors.^11^ The COVID-19 pandemic has caused a much greater appreciation of the frequency and disproportionately deleterious effects of secondary stressors. Also, notions of organisational justice can markedly influence the motivation and well-being of staff.^33^

Secondary stressors continued and accumulated as waves 1 and 2 of the pandemic continued. Converting some hospitals to entirely COVID-19 environments has had huge implications for staffing, especially where it involved closing departments. Where hospital departments were reconfigured, often more than once, there has been a strain on staff trying to communicate these changes effectively to all concerned. Providing psychological or other well-being services was also not always clearly communicated, resulting in staff being unable to gain access, and there were disparities in provision across hospitals, which left some staff underserved. Over the summer of 2020, as cases dropped in the UK, support services for staff were steadily withdrawn, ‘wellness rooms’ converted back to their original use and psychologists stood down from providing extra services. At the time of writing this article, cases were very high in the UK, but our observation is that psychosocial support in workplaces is not being provided at the rate it was earlier in the pandemic.

Throughout the pandemic, there has been a huge volume of necessary communication, especially early on. This resulted in staff having to try to keep up to date by email, telephone calls and social media (for example, WhatsApp) to keep abreast of developments. Given that this occurs at times when staff are on the highest alert and least able to process new information, and less able to switch off, sleep or take breaks, there is additional stress and distress with regard to being informed and remaining safe. The pressure on more senior staff to manage not only their own information burden and uncertainty, but also that of their more junior colleagues, was immense. There is now anecdotal information suggesting that the burden of stress has passed to managers and the more senior clinical staff as time has passed.

Many staff in the UK have been redeployed in the most acute phases of the response to the pandemic, some voluntarily and some less so, with whole teams redeployed either within their own hospital or to other hospitals or specialist COVID-19 centres. Although we have heard reports of very positive experiences, especially regarding the opportunity to learn new skills and the efforts made to create functioning teams very rapidly, there have also been some very difficult experiences. We have heard from redeployed colleagues that feeling insufficiently skilled was a great source of stress to them, especially in such high-stakes situations as intensive care units. Equally, those staff who have been redeployed, or whose workloads were put on hold or redistributed during the most acute phases, experienced feelings of guilt and worry about the work left undone. They were also unable to experience the good feelings that would normally have come from undertaking the work they were trained to do. There was a huge need for education and training in the light of redeployment, but not always much clarity about who would provide it. Local education teams in hospitals had to rapidly develop new training programmes based on local need and quickly provide them for growing groups of staff, not all of whom were familiar with each other or the environment. Grants were given for exploring innovative ways to train people to communicate more effectively when wearing full PPE. Training hubs such as the London Transformation and Learning Collaborative (see https://www.e-lfh.org.uk/programmes/london-transformation-and-learning-collaborative-ltlc/) aim to offer online training for a wide range of critical care skills, building on the learning from earlier phases of COVID-19.

The kinds of stressors that we have identified in our continuing contacts with colleagues resonate with those reported by Kisely et al.^2^ In addition, we draw attention to a trope that grew during the first wave in the UK: celebrating healthcare staff as heroes. Later, this description was extended to many other essential workers. They were amplified in the media, resulting in a weekly occurrence of public recognition that lasted for 10 weeks. Although at first glance this was positive, we feared at the time that this ritual might result in some staff struggling to allow themselves to process their emotions in relation to the pandemic. In reality, many of them were and are afraid and angry; being portrayed as a hero made these emotions seem somehow aberrant. These celebrations have not resurfaced during the second wave despite some attempts to revive the practice. On the other hand, staff are detrimentally experiencing the claims of a small number of deniers and conspiracy theorists who assert that the extent and seriousness of COVID-19 have been grossly exaggerated. Some point to empty hospital corridors in new hospitals as evidence of malfeasance. These claims are terribly hurtful to staff who are putting their lives on the line during the pandemic, and add to the stress they are experiencing. In reality, a recent survey of the Fellows of the Royal College of Physicians (RCP) indicates that ‘more than 1 in 4 doctors have sought mental health support during the pandemic’.^34^

In summary, the research and anecdotal evidence to date indicates that healthcare staff face a mix of stressors of kinds that are recognised in other infectious disease outbreaks. Although it is easy to focus on the primary stressors relating to fear of contracting or transmitting a highly dangerous disease, there are secondary stressors that should be more avoidable or changeable.^11,32^ Although NHS England and NHS Improvement (NHSEI) have admirably stepped up their support for their staff, we have two concerns. First is the tendency not to afford secondary stressors the focus they require, especially because the culture of care for staff taken into the outbreak has been based on failures to recognise these matters. Second is that the enhanced care for healthcare staff may be allowed to lapse once the staff ‘cease to be heroes’. However, importantly, the People Plan published by NHSEI at the end of July 2020 does promise to continue some of the benefits offered to staff throughout the pandemic, and to focus on improving the culture of the NHS in England.^35^

### Mobilisation and deterioration of social support after disasters

In 2009, Kaniasty and Norris recognised two prominent pathways of collective behaviour in responses to major incidents. They are the support mobilisation and support deterioration paths.^36^ These paths relate to the actual support received by people affected and their perceptions of social support. The former is most apparent in the early days of people responding to serious events, whereas the latter is particularly evident as support declines later on.

The mobilisation path is usually seen to operate in communities affected by all kinds of disaster. It is based on actual social support expanding rapidly as people come together and share social identities in the face of what they perceive as a common fate. It is characterised by emergent altruistic communities, democracy of distress, heightened solidarity and reduced levels of interpersonal conflict. There appears to us little doubt that we witnessed this path during the first wave of the pandemic in the UK. We are struck by its clear emergence in such a different emergency as presented by this pandemic.^37^ One of us (R.W.) has been involved in researching how long these effects last after common disasters such as flooding. After single-event disasters, the mobilisation of social support is characteristically followed several months later by the effects of the deterioration path, and we were forcibly struck by its appearance in the pandemic at a time that coincided with the decline of the first wave. This path is characterised by altruism and camaraderie slowly reducing, and being replaced by disillusionment as the harsh realities become plain. The opinion of Kaniasty and Norris is that this occurs because people's expectations of being offered social support reduce as tight social identities in groups loosen over time; their perceptions of being supported diminish because expectations of support are less embedded in teams and communities.^36^ In the UK, we witnessed these developments from June 2020 onward.

The deterioration path that occurred through the summer of 2020 was evident in many communities and, to perhaps a lesser extent, in some NHS staff teams. Those effects are still evident 9 months later, and there does not appear to have been such a widespread spontaneous re-emergence of the support mobilisation path. Arguably, NHS staff, who no longer receive such visible signs of public esteem, including the public providing cakes and meals in addition to good wishes, were under greater pressure during the second wave than the first wave. Nonetheless, we observe that the vaccination programme in the UK is accompanied by great public optimism. But, as a recent survey of doctors shows, ‘The second wave of coronavirus is undoubtedly hitting the NHS far harder than the first, with three-quarters of respondents finding this second wave either slightly or much busier compared to the peak in April [2020], and 56% very concerned about the impact of rising COVID-19 admissions on their organisation's capacity to deliver safe and effective care’.^34^

Consequently, sustaining teams has assumed a much greater focus for the support services as time has passed. What stands out for us is that research on single-event disasters appears to apply to the existential threat posed by the longer-term wave emergency of this pandemic.

## What healthcare services might hold on to in the post-pandemic era

### Positive lessons learned about caring for staff in the response to COVID-19

We have observed many positive endeavours to improve care for healthcare staff in the response to the pandemic, and some vital successes. Early on, we produced a document to inform preparations as the work of the NHS in the UK was diverted in response to the need to focus on caring for people who have COVID-19.^38^ In it, we urged managers and senior leaders to enable staff to face honestly and straightforwardly the realities of the risks they face, while being willing to hear about problems such as access to PPE and equipment, which fall into the domain of secondary stressors.

During the first wave, we know, anecdotally, that team-working and leadership appeared and were welcomed by staff as an alternative to organising staff on the basis of duty rotas. The levels of altruism were enormous. Staff took time to listen to, and care for each other. We counsel taking steps to avoid losing these important aspects of the cultures in which we work. There were encouraging developments with regard to staff training, in which some staff, who traditionally could not attend training because they were unable to get time away from the shopfloor, have attended training during the pandemic. It is clear that, when necessary, it is possible to liberate staff to enable them to attend training, and this should continue. Attention should be sustained regarding maintaining the coherence of teams in the face of pressures on senior clinicians and health service managers, and continuing public disillusionment. This is an important challenge to the effects of the deterioration path.

We have learned that there are many ways in which ‘being on the front line’ is defined, and most services have faced dealing with COVID-19 in their midst.^39^ Clearly, staff who are not ‘on the front line’ have been making vital contributions, and it is imperative to avoid the splitting that comes from valuing some people's work over the contributions of others. We have also seen, for example, that there are important roles for staff who are, rightly, being shielded. Their effective employment and using their talents well are vital to sustaining services as well as to their morale and well-being.

There has been an increase in the use of virtual consultations, which does bring many advantages in terms of access to patients who might otherwise struggle to attend clinics. A unique issue in out-patient psychiatry during the pandemic is that, if patients were seen in consulting rooms, they would be wearing face masks, precluding appreciation of facial expressions. However, with the adoption of telepsychiatry, no masks are worn and psychiatrists are able to note facial expressions.

But despite these advantages, virtual or remote working also creates a level of challenge and anxiety around managing people who have chronic conditions, and the concern that some worrying symptoms might be missed in otherwise healthy patients. This increases the burden of stress on staff, especially those working in communities, who would normally have seen patients in person. Thus, being able to engage in meaningful work cannot offset the feelings of worry, loneliness, isolation and frustration that arise from being away from colleagues and patients. The informal social interactions that would have taken place, for example, at the beginning and ending of meetings, or walking to and from meetings and clinics, have also been lost, and the online format means that meetings are far more transactional in nature than they were before. This brings us onto recognising the importance to staff and, therefore, to patients and services, of their families and friends. Supporting staff who work with patients is a vital contribution that families and friends make, yet that is seldom recognised in healthcare.

What becomes clear is that when people work as members of an organisation like the NHS, there is a complex relationship between the organisation's reputation and the reality of attempting to provide services. The elements of myth and magic associated with an organisation that has provided excellent healthcare for 70 years and is free at the point of delivery in the UK, are only amplified in a high-stakes situation like this pandemic. We have seen the talk of NHS ‘heroes’ and ‘angels’, the enormous field hospitals equipped at speed, the media reporting of the brave doctors and nurses on the front line, risking their lives. That was much less evident from about June 2020 onward in the UK. However, staff in the hospitals know that the reality is very different: there have been difficulties in supplying sufficient PPE for staff,^25,27^ and they have faced their colleagues rapidly falling sick and certain groups of colleagues getting sicker than others.^40,41^ In parallel, there were media allegations that the ‘real’ stories could not be told because some staff were not allowed to tell the truth about what they were witnessing in their workplaces.^42^

## The agenda for healthcare staff now and in the future

**Fig. 1:**
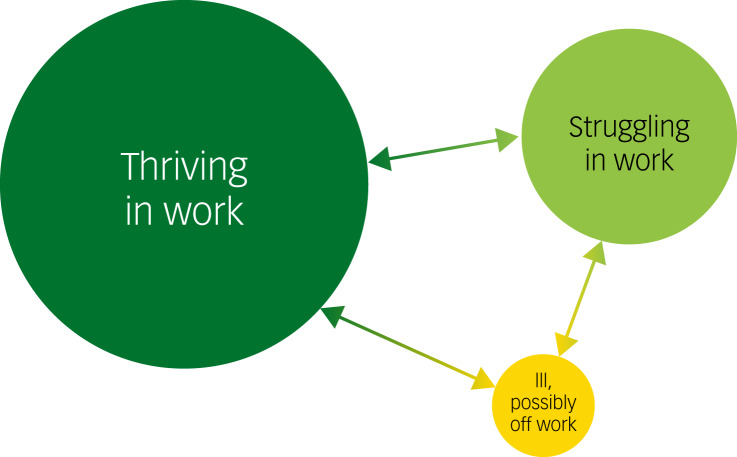
Three phases people experience in work (reproduced from Stevenson and Farmer,^43^ under Open Government Licence v3.0.).

Just before the pandemic began, Williams and Kemp published an introductory paper that links to work being done by the Faculty of Pre-Hospital Care in the Royal College of Surgeons of Edinburgh (FPHC) to care better for healthcare staff in the UK.^11^ The paper overviews some of the findings from the FPHC's Psychosocial and Mental Health Programme. The approach it espouses builds on work published in the review by Stevenson and Farmer (Fig. 1).^43^ The programme recognises that most people in work may be flourishing or struggling, and that a smaller proportion may be ill while at work. In reality, there is a dynamic interchange within these three groups.

Fig. 1 is reproduced from the report, and it frames three challenges.^11^ They are how to assist employees who are thriving at work to continue to do so and, indeed, to flourish (the well-being agenda); how to support staff who are struggling (the psychosocial agenda) and how to enable people who are ill to recover and return to work (the mental health agenda).

We see this approach as being all the more relevant after our experiences with COVID-19. Importantly, it frames tasks for employers to adapt their organisations to reduce sickness and absence levels caused by stress and mental ill health, by improving their engagement with staff and offering positive and supportive workplace cultures.

We espouse the three objectives of Stevenson and Farmer in creating a renewed approach to caring for staff that balances their employers’ responsibilities with their responsibilities to themselves. People who work on well-being have long desired to create a culture within workplaces in which employees thrive at work and are able to support staff who are struggling, and enable those who have become ill to recover and return; until this time, these changes have appeared insurmountable, but perhaps the pandemic gives us the opportunity to rethink as we reconfigure.

### The well-being agenda

We recommend that staff should be offered opportunities to sign up to a programme of continuing personal and professional development that persists after the pandemic is controlled. Similarly, there should be an accent on developing/training teams and places of employment to ensure that they are fit to support staff, and respond to their needs arising from both primary and secondary stressors. In these ways, staff should be assisted to remain flourishing contributors. Schwartz Rounds are interactive approaches that bridge between the well-being and psychosocial agendas.^44^

### The psychosocial agenda

The RCP survey indicates that, although a third of respondents reported felt supported (35%) and determined (37%), the majority of doctors (64%) felt tired or exhausted, and many were worried (48%).^34^ Inequities between staff groups, which existed before COVID, have been thrown into more stark relief by the pandemic. As was the case in previous pandemics and epidemics, healthcare workers have been significantly affected, both in terms of psychological impact and morbidity and mortality.^1,2,45–47^ A new development in the COVID-19 pandemic is that Black and minority ethnic staff groups have suffered a far higher mortality rate than other ethnic groups.^13^ Findings such as these frame the essence of the psychosocial agenda as aiding people who are struggling to return to good well-being. In this context, we talk here of interventions rather than treatments because people who are struggling are not ill, but are nonetheless suffering. Supporting interventions should be based on the principles of psychological first aid and, importantly, include peer support.^15,48–51^

### The mental health agenda

The mental health agenda concerns ensuring that there are effective services that are available to staff whose psychosocial and mental health needs are more serious and might amount to mental ill health. As we write, we are observing the NHSEI implementation of a network of mental health hubs across England, to ensure that the psychosocial and mental healthcare needs of healthcare staff are responded to in timely and effective ways. Their contributions are likely to include offering assessments and access to psychological and psychiatric treatments when they are required.

### Dealing with recovery

As COVID-19 recedes, hopefully in the summer of 2021 (but not imagining that it will be gone by then), we have an opportunity to learn from these recent experiences and consider the lessons we might take forward. We have known for some time that there should be a culture change in healthcare, both in the UK and the USA.^2,52^ A key challenge that is more specific to the effects of the COVID-19 pandemic on staff is attending to the matters that are included in Table 1.

**Table 1:** Matters that require attention as the challenge of COVID-19 begins to settle

Rest and recuperation - staff need time to recover their energy, reflect and prepare for repeated or new challenges Credit staff with the positives, enable them to own the successes and carry the positive aspects forward Enable staff to come to terms with the moral frustration, distress and injury they may well be experiencing Recognise and deal with the secondary stressors that have affected staff during the crisis (apparently small matters such as car parking, staff accommodation, provision of food and facilities for showering are experienced as major items by staff) Wind-down for staff who have returned to practice or work in the NHS from, for example, retirement Training Catch-up training for early entrants (people who entered NHS services ahead of their anticipated graduation dates) Meeting the needs of trainees, and especially trainees moved elsewhere during the crisis, and for restoration of their disrupted training Restore organisations and regenerate teams Avoid collision of recovery with deferred elective care, waiting lists, winter pressures, etc. Deal with pre-COVID-19 legacy issues in which many staff were and continue to be affected by secondary stressors Be prepared for the possible lengthy duration of the needs of staff Truth and reconciliation style processes to allow grievances to be heard and processed by the healthcare professional community in a safe and tested format

Reproduced with permission (© R. Williams, 2020. All rights reserved). Table 1 is based on a webinar given by R Williams to the Royal College of Psychiatrists 2020.^53^ NHS, National Health Service.

An issue that should be addressed if teams are to function well in the future is what is known colloquially as ‘survivor guilt’. Staff who have had to stay away from work, perhaps because they were shielding, have reported feeling guilty, and their reintegration in the workplace, when it occurs, must be supported. For some working remotely, it is not only guilt, but frustration and even anger for their efforts not being appropriately appreciated.^54^ Other staff who were at work report guilt at having to postpone assessing and treating patients who may have other conditions. It is vital for leaders to support everyone in managing their reactions to one another when teams change and reconfigure.

Some staff have contracted COVID-19 and have their own stories to tell. It is important to learn lessons from these experiences about the practicalities, such as providing food and lodging, or rapid testing, as well as the ways in which we might communicate usefully with colleagues to support them in such frightening circumstances. Open discussions of the feelings that have arisen with regard to colleagues, the pandemic and their work are likely to be useful in this regard. Acknowledging and responding to the commonality of staff's experiences in the pandemic, the nature, severity and effects of their distress and other feelings about the threat, exposure, fatigue and self-care are extremely important. Improving communication within teams, team dynamics and concerns are central to sustaining culture change in organisations. Staff need to know that there will be continued support, in whatever format works best for them, beyond the current pandemic.

In light of the current situation, various colleges are working together to understand how flows of patients through healthcare systems can work in ways that keep staff and patients safe. An example of this is provided by the Royal College of Emergency Medicine and the College of Paramedics, which are collaborating to create safer emergency departments and avoid the long-standing overcrowding.^55^ An end to ‘corridor medicine’ would benefit the well-being of patients and emergency department staff. There are many other changes we might take forward, but the simple truth is that we must do things differently in the future if healthcare workers are to thrive.

There must be willingness to begin culture changes and the process of making these much-needed advances permanent rather than dropping them once the crisis appears to have passed.^56^ If healthcare continues to focus on throughput of patients with little time for appropriate care, and still cannot meet its own targets for screening, diagnosing and treating the more usual suspects of heart disease, diabetes and cancer, then little else can change. We argue that moving to examine the agenda for improving quality of care could speed up rather than slow down processes for moving patients through healthcare systems. Certainly technological advances have shown that there are new ways of doing things, which were previously valued, and online supportive reflective practice groups, and clinical ethics committees are proliferating.^57^ Threats to change come from predictable quarters, a perfectly human reluctance on the part of many people to embrace change at all, change fatigue, COVID-19/pandemic fatigue,^58^ financial constraints and the relentless nature of medicine, which leaves little time for thoroughgoing change projects. Despite the roll out of vaccines for COVID-19 over 2021, it will be important to remember that staff need rest, recuperation and time to process their experiences, and be expected to carry on when they have immunity.^34^

The problem with a narrative of heroes, angels and brave front-line workers, which so caught the attention of the media, is that it perpetuates the myth of healthcare workers as almost superhuman in their resilience and fortitude, even as we have a parallel narrative of burned-out staff, at risk of post-traumatic stress disorder.^59^ This binary leaves no place for staff to situate themselves as ordinary humans doing a challenging job. As long as healthcare professionals are depersonalised in this manner, there will not be space for the culture change that must take place to allow space for the natural ebb and flow of illness, including overdue emphasis on mental health,^38^ the parity of esteem agenda and return to fitness.^60^ In her paper on heroism in disasters and major incidents, Eyre points out that this moralistic approach to people doing their jobs raises questions about what happens in dangerous situations in which employees’ health is at risk, and prompts us to consider the moral architecture of healthcare organisations and the role of employers in protecting their staff.^61,62^ This issue has been front and centre in the COVID-19 crisis, as so many staff have perceived themselves as practising without adequate PPE.^63^

Unlike sports teams, there is no second squad of healthcare professionals waiting on a bench to substitute those on duty now so that the team on the pitch can rest. Even as the second wave is proceeding in the UK and we recognise that normal service was already overwhelmed, staff are all too aware that routine operations, diagnostic procedures and reviews have been reduced substantially as the NHS in the UK has been repurposed. The junior doctors’ strike in the UK was probably the most well-known moment in which the workload of healthcare workers was brought to the fore. Structural changes in hospitals with expanding patient numbers and finite estates meant that, for example, on-call rooms in which staff could rest had been steadily disappearing. In the pandemic, rotas have been turned upside down to manage workload. The lack of understanding of the natural course of COVID-19 infection gives us an interesting metaphor with which to work. This illness has behaved in unexpected ways, treatment has been a challenge and it appears to leave many sequelae affecting many organ systems – including the brain and central nervous system in some people – that are poorly understood.^64^

Fatigue has been widely reported as one of the most debilitating and persistent symptoms of COVID-19.^65^ The sheer length of time that some people are taking to recover from COVID-19 is a timely reminder that for some illnesses, a period of convalescence is necessary. This was well-understood by our forbearers, but, with the advent of ever more sophisticated treatments, vaccinations and the eradication of some serious contagious diseases, we have allowed ourselves to forget. This period, during which health and strength should gradually return, was characterised by rest, certain foods deemed suitable for building strength, sleep, gentle exercise, the removal of sources of overstimulation and above all, a gradual return to normal life.^66^ In the past, practitioners understood that a failure to properly recuperate from an illness might result in patients becoming ill again, and that some people would never regain their former vigour. With regard to COVID-19, we find ourselves challenged in many ways, not least of which is how to allow rest for a workforce that was already overstretched.

We have an opportunity to recognise that the nature of the COVID-19 pandemic and the accumulation of years of rapid change have left staff and services exhausted and depleted.^34,67^ We must find a way to allow staff to have a convalescent period, a gradual recovery of health and strength, during which we slow the pace a little and review what is working or not working in supporting the psychosocial care for staff, and to make concerted efforts to listen to feedback and keep what is good.

There is good evidence that most people are likely to recover from the psychosocial effects of working during the pandemic with enough social support and, where indicated, more formal interventions and treatment. Psychiatry and psychology are already convinced of the therapeutic value of being able to tell one's story, especially to those who have had similar experiences: this type of social support helps to reduce feelings of isolation, which compound loneliness and trauma. Equally, we know that the telling stories can be uncomfortable and can challenge institutions, especially if there is the implication that mistakes were made. But if we can allow ourselves to listen to the stories of staff who have been working in healthcare through the pandemic, we can ensure that their experiences were not for nothing.

## Conclusions

Despite all we have learned from prior disasters and infectious outbreaks, the COVID-19 pandemic makes us focus again on the need to rethink and restructure the healthcare culture in the NHS to ensure the long-term well-being of healthcare providers. There is much that has been and is being done to care for staff during the pandemic, and much of that should be adapted and taken into healthcare systems after the crisis passes.

We recommend that we should revisit these matters in a year's time, to see what healthcare services in the UK and other countries have done for both healthcare workers and healthcare organisations to ensure personal and system-wide improvements in culture and care. Furthermore, given the potential for future widescale infectious disease outbreaks, we recommend that healthcare systems should periodically revisit the important topics we have discussed here, to see that we have truly learned and acted upon the lessons, because we can ill afford to deal with similar demanding matters in the future.

## References

[1] Sirois FM, Owens J. Factors associated with psychological distress in health-care workers during an infectious disease outbreak: a rapid systematic review of the evidence. Front Psychiatry 2020; 11: 589545. 10.3389/fpsyt.2020.589545 PMC7876062 33584364

[2] Kisely S, Warren N, McMahon L, Dalais C, Henry I, Siskind D. Occurrence, prevention, and management of the psychological effects of emerging virus outbreaks on healthcare workers: rapid review and meta-analysis. BMJ 2020; 369: m1642. 32371466 10.1136/bmj.m1642 PMC7199468

[3] Sykes C, Borthwick C, Baker E. Mental Health and Wellbeing in the Medical Profession and British Medical Association. British Medical Association, 2019 (https://www.bma.org.uk/media/1362/bma-mental-health-and-wellbeing-medical-profession-full-report-oct-2019.pdf).

[4] NHS England. Simon Stevens Announces Major Drive to Improve Health in NHS Workplace. NHS England, 2015 (https://www.england.nhs.uk/2015/09/nhs-workplace/).

[5] Younie L, Swinglehurst D. Creative enquiry and reflective general practice. Br J Gen Pract 2019; 69(686): 446–7. 31467014 10.3399/bjgp19X704969 PMC6715485

[6] Boellinghaus I, Jones FW, Hutton J. The role of mindfulness and loving-kindness meditation in cultivating self-compassion and other-focused concern in health care professionals. Mindfulness 2014; 5: 129–38.

[7] Galloway R. EM-POWER: A Practical Guide to Flexible Working and Good EM Rota Design (Oct 2019). Royal College of Emergency Medicine, 2019 (https://tinyurl.com/y6rn3amq).

[8] Gispen F, Wu AW. Psychological first aid: CPR for mental health crises in healthcare. J Patient Saf Risk Manage 2018; 23(2): 51–3.

[9] Department of Health. NHS Emergency Planning Guidance. Planning for the Psychosocial and Mental Healthcare of People Affected by Major Incidents and Disasters: Interim National Strategic Guidance. Department of Health, 2009 (https://webarchive.nationalarchives.gov.uk/20130129032354/http:dh.gov.uk/en/Publicationsandstatistics/Publications/DH_103562).

[10] Hardacre J, Margetts A. Psychological PPE: Survival Kit for Creating a Safer Culture in the COVID-19 Context. BMJ Leader, 2020 (https://blogs.bmj.com/bmjleader/2020/04/15/psychological-ppe-survival-kit-for-creating-a-safer-culture-in-the-covid-19-context/).

[11] Williams R, Kemp V. Caring for healthcare practitioners. BJPsych Adv 2019; 26(2): 116–28.

[12] Shanafelt T, Ripp J, Trockel M. Understanding and addressing sources of anxiety among health care professionals during the COVID-19 pandemic. JAMA 2020; 323(21): 2133–4. 32259193 10.1001/jama.2020.5893

[13] Public Health England. Disparities in the Risk and Outcomes of COVID-19. Public Health England, 2020 (https://assets.publishing.service.gov.uk/government/uploads/system/uploads/attachment_data/file/908434/Disparities_in_the risk_and_outcomes_of_COVID_August_2020_update.pdf).

[14] Kaufman KR, Petkova E, Bhui KS, Schulze TG. A global needs assessment in times of a global crisis: world psychiatry response to the COVID-19 pandemic. BJPsych Open 2020; 6(3): e48. 32250235 10.1192/bjo.2020.25 PMC7211994

[15] Williams R, Bisson JI, Kemp V. Healthcare planning for community disaster care. InTextbook of Disaster Psychiatry (2nd edn) (eds RJ Ursano, CS Fullerton, L Weisaeth, B Raphael): 244–260. Cambridge University Press, 2017.

[16] World Health Organization. WHO Calls for Healthy, Safe and Decent Working Conditions for all Health Workers amidst COVID-19 Pandemic. World Health Organization, 2020 (https://tinyurl.com/yab7cmn8).

[17] United Nations. Policy Brief: COVID-19 and the Need for Action on Mental Health. United Nations, 2020 (https://tinyurl.com/ycq4p7f7).

[18] Lock S, Rubin GJ, Murray V, Rogers MB, Amlôt R, Williams R. Secondary stressors and extreme events and disasters: a systematic review of primary research from 2010-2011. PLoS Curr 2012; 4: ecurrents.dis.a9b76fed1b2dd5c5bfcfc13c87a2f24f. 10.1371/currents.dis.a9b76fed1b2dd5c5bfcfc13c87a2f24f PMC3492002 23145350

[19] Jameton A. Nursing Practice: The Ethical Issues. Prentice-Hall, 1984.

[20] Litz BT, Stain N, Delaney E, Moral injury and moral repair in war veterans: a preliminary model and intervention strategy. Clin Psychol Rev 2009; 29: 695–706. 19683376 10.1016/j.cpr.2009.07.003

[21] Shay J. Moral injury. Psychoanalitic Psychol 2014; 31(2): 182–91.

[22] Campbell D. *More Than 1,000 UK Doctors Want to Quit NHS Over Handling of Pandemic. The Guardian*, 2020 (https://www.theguardian.com/society/2020/sep/05/more-than-1000-doctors-want-to-quit-nhs-over-handling-of-pandemic).

[23] The New York Times. *Coronavirus in the U.S.: Latest Map and Case Count.* The New York Times, 2021 (https://www.nytimes.com/interactive/2020/us/coronavirus-us-cases.html).

[24] White DB, Lo B. A framework for rationing ventilators and critical care beds during the COVID-19 pandemic. JAMA 2020; 323(18): 1773–4. 32219367 10.1001/jama.2020.5046

[25] Mantelakis A, Spiers HVM, Lee CW, Chambers A, Joshi A. Availability of personal protective equipment in NHS hospitals during COVID-19: a national survey. Ann Work Expo Health 2021; 65(1): 136–40. 10.1093/annweh/wxaa087 PMC7499547 32914175

[26] World Health Organization. Shortage of Personal Protective Equipment Endangering Health Workers Worldwide. World Health Organization, 2020 (https://www.who.int/news/item/03-03-2020-shortage-of-personal-protective-equipment-endangering-health-workers-worldwide).

[27] Iqbal MR, Chaudhuri A. COVID-19: results of a national survey of United Kingdom healthcare professionals’ perceptions of current management strategy - a cross-sectional questionnaire study. Int J Surg 2020; 79: 156–61. 32447002 10.1016/j.ijsu.2020.05.042 PMC7241367

[28] Hampton T, Crunkhorn R, Lowe N, Bhat J, Hogg E, Afifi WS, The negative impact of wearing personal protective equipment on communication during coronavirus disease 2019. J Laryngol Otol 2020; 134(7): 577–81. 32641175 10.1017/S0022215120001437 PMC7387788

[29] Mitchell A, Cummins T, Spearing N, Adams J, Gilroy L. Nurses’ experience with vancomycin-resistant enterococci (VRE). J Clin Nurs 2002; 11(1): 126–33. 11845749 10.1046/j.1365-2702.2002.00560.x

[30] Dean E. COVID-19: End of Life Care and the Lessons of the First Wave. Nursing Standard, 2020 (https://rcni.com/nursing-standard/newsroom/analysis/covid-19-end-of-life-care-and-lessons-of-first-wave-168201).

[31] Collini A, Parker H, Oliver A. Training for difficult conversations and breaking bad news over the phone in the emergency department. Emerg Med J 2021; 38: 151–4. 10.1136/emermed-2020-210141 33273038

[32] Williams R, Ntontis E, Alfadhli K, Drury J, Amlôt R. A social model of secondary stressors in relation to disasters, major incidents and conflict: implications for practice. *Int J Disaster Risk Reduct* 2021; 63: 102436.

[33] Latham GP, Pinder CC. Work motivation theory and research at the dawn of the twenty-first century. Annu Rev Psychol 2005; 56: 485–516. 15709944 10.1146/annurev.psych.55.090902.142105

[34] Royal College of Physicians. More Than 1 in 4 Doctors Have Sought Mental Health Support during the Pandemic. Royal College of Physicians, 2021 (https://tinyurl.com/y2co83ul).

[35] NHS England and NHS Improvement. We Are the NHS: People Plan for 2020/2021 – Action for Us All. NHS England and NHS Improvement, 2020 (htpps://www.england.nhs.uk/ournhspeople/).

[36] Kaniasty K, Norris FH. Distinctions that matter: received social support, perceived social support and social embeddedness after disasters. In Mental Health and Disasters (eds Y Neria, S Galea, FH Norris): 175–200. Cambridge University Press, 2009.

[37] Ntontis E, Drury J, Amlôt R, Rubin GJ, Williams R, Morales PJS. Collective resilience in the disaster recovery period: emergent social identity and observed social support are associated with collective efficacy, wellbeing and the provision of social support. Br J Soc Psychol 2021; 60(3): 1075–95. 10.1111/bjso.12434 33340132

[38] Williams R, Murray E, Neal A, Kemp V. Top Ten Messages for Supporting Healthcare Staff during the COVID-19 Pandemic. Royal College of Psychiatrists, 2020 (https://tinyurl.com/wm7e3pn).

[39] NHS Providers. Spotlight on the Impact of COVID-19 on Mental Health Trusts in the NHS. NHS Providers, 2020 (https://tinyurl.com/y69arwaz).

[40] Cook T, Kursumovic E, Lennane S. *Exclusive: Deaths of NHS Staff from COVID-19 Analysed*. HSJ, 2020 (https://www.hsj.co.uk/exclusive-deaths-of-nhs-staff-from-covid-19-analysed/7027471.article).

[41] Cook TM, Kearney L, Kursumovic E, Lennane S. Younger Female NHS Staff Death Rate Is Double That of Non-Staff. HSJ, 2020 (https://www.hsj.co.uk/coronavirus/younger-female-nhs-staff-death-rate-is-double-that-of-non-staff/7027560.article).

[42] Johnson S. *NHS Staff Forbidden from Speaking Out Publicly about Coronavirus. The Guardian*, 2020 (https://www.theguardian.com/society/2020/apr/09/nhs-staff-forbidden-speaking-out-publicly-about-coronavirus).

[43] Stevenson D, Farmer P. Thriving at Work: The Stevenson/Farmer Review of Mental Health and Employers. Department for Work and Pensions and Department of Health and Social Care, 2017 (https://assets.publishing.service.gov.uk/government/uploads/system/uploads/attachment_data/file/658145/thriving-at-work-stevenson-farmer-review.pdf).

[44] Montgomery J, Haslam SA, Neal A, Williams R. Commentaries on core themes in section 2. In Social Scaffolding: Applying the Lessons of Contemporary Social Science to Health and Healthcare (eds R William, V Kemp, SA Haslam, C Haslam, KS Bhui, S Bailey): 123–8. Cambridge University Press, 2019.

[45] Brooks SK, Webster RK, Smith LE, Woodland L, Wessely S, Greenberg N, The psychological impact of quarantine and how to reduce it: rapid review of the evidence. Lancet 2020; 395: 912–20. 32112714 10.1016/S0140-6736(20)30460-8 PMC7158942

[46] Fernandez R, Lord H, Halcomb E, Moxham L, Middleton R, Alananzeh I, Implications for COVID-19: a systematic review of nurses’ experiences of working in acute care hospital settings during a respiratory pandemic. Int J Nurs Stud 2020; 111: 103637. 10.1016/j.ijnurstu.2020.103637 PMC7206441 32919358

[47] Bandyopadhyay S, Baticulon RE, Kadhum M, Alser M, Ojuka DK, Badereddin Y, Infection and mortality of healthcare workers worldwide from COVID-19: a systematic review. BMJ Glob Health 2020; 5(12): e0003097. 10.1136/bmjgh-2020-003097 PMC7722361 33277297

[48] NHS England. Psychological First Aid in Emergencies Training for Frontline Staff and Volunteers. NHS England and NHS Improvement, 2020 (https://www.gov.uk/government/news/psychological-first-aid-in-emergencies-training-for-frontline-staff-and-volunteers).

[49] Sidhu J, Williams R, Kemp V. A Quick Guide to Peer Support: How Doctors Can Support Their Peers during the COVID-19 Pandemic. Royal College of Physicians, 2020 (https://www.rcplondon.ac.uk/projects/peer-support-new-rcprcpsych-guidance-values-principles-and-practice).

[50] Williams R, Kemp V. The Nature of Peer Support. Royal College of Physicians and Royal College of Psychiatrists, 2020 (https://www.researchgate.net/publication/341542226_The_nature_of_peer_support).

[51] Williams R, Kemp V, Stokes S, Lockey D. Peer Support. Royal College of Surgeons of Edinburgh, 2020 (https://fphc.rcsed.ac.uk/media/2841/peer-support.pdf).

[52] Hartzband P, Groopman J. Physician burnout, interrupted. N Engl J Med 2020; 382(26): 2485–7. 32356624 10.1056/NEJMp2003149

[53] Williams R. Caring for Healthcare Staff: Using Lessons from the Pandemic to Improve Care for NHS Staff When the Pandemic Resolves. Royal College of Psychiatrists Webinar 2020 (URL no longer available).

[54] Chattopadhyay I, Davies G, Adhiyaman V. The contributions of NHS healthcare workers who are shielding or working from home during COVID-19. Future Health J 2020; 7(3): e57–9. 10.7861/fhj.2020-0096 PMC7571755 33094257

[55] Royal College of Emergency Medicine. RCEM Launches New Campaign to End Corridor Care as Data Shows more than 100,000 Patients Waiting over 12 hours in A&Es This Winter. Royal College of Emergency Medicine, 2020 (https://tinyurl.com/yy2dcgdn).

[56] Hannan R. Why We Shouldn't Use War Metaphors to Talk about Healthcare. Royal Society for Arts, Manufactures and Commerce, 2020 (https://www.thersa.org/discover/publications-and-articles/rsa-blogs/2020/05/war-language-healthcare).

[57] Roberts M. Balint groups: a tool for personal and professional resilience. Can Fam Physician 2012; 58(3): 245–7. 22423015 PMC3303639

[58] Gerada C, Walker C. Covid Fatigue Is Taking an Enormous Toll on Healthcare Workers. The BMJ Opinion, 2020 (https://tinyurl.com/yygol3pm).

[59] Carmassi C, Foghi C, Dell'Oste V, Cordone A, Bertelloni CA, Bui E, PTSD symptoms in healthcare workers facing the three coronavirus outbreaks: what can we expect after the COVID-19 pandemic. Psychiatry Res 2020; 292: 113312. 32717711 10.1016/j.psychres.2020.113312 PMC7370915

[60] Bailey S. Chapter 8: parity of esteem for mental health. In Social Scaffolding: Applying the Lessons of Contemporary Social Science to Health and Healthcare (eds R Williams, V Kemp, SA Haslam): 66–71. Cambridge University Press, 2019.

[61] Eyre A. The making of a hero: an exploration of heroism in disasters and implications for the emergency services. Int Fire Serv J Leadersh Manage 2014; 8: 7–16.

[62] Williams R, Kemp V, Neal A. Compassionate care: leading and caring for staff of mental health services and the moral architecture of healthcare organisations. In Management for Psychiatrists (4th edn) (eds D Bhugra, S Bell, A Burns): 377–402. RCPsych Publications, 2016.

[63] Jones-Berry S. COVID-19: RCN Demands Action over ‘Unconscionable’ Lack of PPE. Nursing Standard, 2010 (https://tinyurl.com/y38t5o83).

[64] Taquet M, Geddes JR, Husain M, Luciano S, Harrison PJ. 6-month neurological and psychiatric outcomes in 236379 survivors of COVID-19: a retrospective cohort study using electronic health records. Lancet Psychiatry 2021; 8(5): 416–27. 33836148 10.1016/S2215-0366(21)00084-5 PMC8023694

[65] Perrin R, Riste L, Hann M, Walther A, Mukherjee A, Heald A. Into the looking glass: post-viral syndrome post COVID-19. Med Hypotheses 2020; 144: 110055. 32758891 10.1016/j.mehy.2020.110055 PMC7320866

[66] Cole WH, Keeton RW, Calloway NO, Glickman N, Mitchell HH, Dyniewicz J, Studies in postoperative convalescence. Ann Surg 1947; 126(4): 592–609. PMC1803419 17859018

[67] West M, Bailey S. Recovery and then Renewal: The Innovation Imperative for Health and Care. The Kings Fund, 2021 (https://www.kingsfund.org.uk/blog/2021/01/recovery-and-then-renewal-innovation-health-and-care-covid-19).

